# Effects of age and gender on body composition indices as predictors of mortality in middle-aged and old people

**DOI:** 10.1038/s41598-022-12048-0

**Published:** 2022-05-12

**Authors:** Chin-Sung Chang, I-Ting Liu, Fu-Wen Liang, Chia-Chun Li, Zih-Jie Sun, Yin-Fan Chang, Ting-Hsing Chao, Chih-Hsing Wu

**Affiliations:** 1 Department of Family Medicine, National Cheng Kung University Hospital, College of Medicine, National Cheng Kung University, 138 Sheng-Li Road, Tainan, 70428 Taiwan; 2 Department of Family Medicine, E-DA Hospital, Kaohsiung, Taiwan; 3 Department of Geriatric Medicine, E-DA Hospital, Kaohsiung, Taiwan; 4 Department of Internal Medicine, National Cheng Kung University Hospital, College of Medicine, National Cheng Kung University, Tainan, Taiwan; 5 Department of Public Health, Kaohsiung Medical University, Kaohsiung, Taiwan; 6 Institute of Gerontology, College of Medicine, National Cheng Kung University, Tainan, Taiwan

**Keywords:** Health care, Medical research, Risk factors

## Abstract

To determine whether body composition indices interact with age and gender as a predictor of all-cause mortality, 1200 participants at least 40 years of age were recruited in 2009 and 2010. A multi-frequency bioelectrical impedance analysis device was used to measure each participant’s body composition indices, including the fat mass index (FMI), fat free mass index (FFMI), skeletal muscle mass index (SMMI), and visceral fat area index (VFAI). A baseline questionnaire was used to collect demographic information about lifestyle habits, socioeconomic status, and medical conditions. All claimed records of death from 2009 to 2018 in the National Health Insurance Research Databank were identified. The all-cause mortality rate was 8.67% after a mean follow-up period of 5.86 ± 2.39 person-years. The Cox proportional hazard model analysis showed significantly negative associations between FFMI or SMMI with all-cause mortality in the total group and those aged $\geq$ 65 y/o. The FFMI and SMMI were negative predictors of mortality in both genders. The FMI and VFAI were positive predictors of mortality exclusively in females. In conclusion, the SMMI is a better predictor of mortality than the BMI, FMI, and FFMI, especially in older adults. A higher fat mass or visceral fat distribution may predict higher mortality in females.

## Introduction

Obesity has become a pandemic and is now one of the most critical health issues in the world^1^, including in Taiwan, where the prevalence of obesity increased sharply from 11.8% to 22.0% from 1993 to 2014^2^. Obesity is well known to be correlated with many chronic diseases, including hypertension, diabetes, cardiovascular disease, and cancers, and may result in higher mortality rates. Although a J or U-shaped relationship is often observed according to different analyses, whether there is a causal benefit of being overweight or obese in the form of longer survival remains controversial, suggesting a possible obesity paradox, especially in older adults^3,4^. Previous studies have mentioned the relationship between BMI and chronic diseases, but many limitations have been found when exploring their influence on all-cause mortality. These phenomena may be due to the fact that the BMI is a relatively poor estimate of body composition and does not accurately evaluate body fat mass and lean mass in the human body^5^. Therefore, disclosing the relationship between body composition indices and all-cause mortality is an emerging issue^6^.

Body composition indices include the BMI, waist circumference (WC), fat mass (FM), fat free mass (FFM), skeletal muscle mass (SMM), and their associated indices^7^. Some studies have utilized direct methods, such as computer tomography (CT), magnetic resonance imaging (MRI), or dual energy X-ray absorptiometry (DXA) to evaluate body composition. Because these instruments are huge, expensive, and involve a risk of exposure to X-rays, they are relatively unavailable for large epidemiological surveys in communities^8^, which has resulted in fewer reports focusing on this issue. Bioelectrical impedance analysis (BIA) and DXA are comparable methods for measuring body composition and are recommended for analyses in Taiwanese and Chinese populations^9,10^. Due to its convenience, a BIA is more popularly used to disclose the relationship between body composition indices and the all-cause mortality rate. Therefore, a BIA was utilized in the present study to assess body composition for the purpose of further investigation.

When the fat mass index (FMI, FM/height^2^) and muscle mass index (MMI) are considered together, increasing adiposity rather than muscle mass has been found to increase survival in older Asian male adults^11^. Among older women in France, the risk of mortality was found to be consistently higher with low adiposity, as represented by a lower BMI and FMI^7^, but no lean mass indicator was associated with a risk of mortality^7,11^. However, there have been studies showing the opposite results, where one study showed a lower appendicular FFM as a predictor of death in older people^12^. A few studies have indicated that higher mortality rates are associated with a lower BMI but with a higher percent of body fat or FM^13,14^. These studies showed inconsistent results for the relationship among different races, genders, and body composition indices with mortality. Thus, more studies should be conducted to clarify the reasons for these conflicting results.

Studies discussing body composition, especially in terms of skeletal muscle mass and visceral fat area and all cause-mortality, are relatively rare and have proven to be inconclusive^7,11,12,14^. These inconclusive results may be contributed by study designs, especially in terms of different age groups, genders, and ethnicities, and few studies have taken biases related to specific health-related behaviors into consideration. Among such indices, the skeletal muscle mass index (SMMI) has been shown to be promising in recent studies^15–17^. Therefore, the aim of this study was to use the BIA method to disclose the relationship between body composition indices and all-cause mortality in order to explore the role of muscle mass, particularly skeletal muscle mass, in all-cause mortality rates among an adult group over 40 years of age, especially in an older group aged over 65 years old in an Asian community cohort living in Taiwan. We hypothesized that body composition indices, especially the SMMI, may contribute heavily to all-cause mortality after adjusting for age, gender, socioeconomic status, co-morbidities, and specific behavioral factors, such as exercise, smoking, and alcohol consumption.

## Results

The all-cause mortality rate in this study was 8.67% (20.09% for the older population) after the mean follow-up period of 5.86 ± 2.39 person-years. The survival group had significantly lower age, Charlson comorbidity index (CCI) scores, fewer smokers, and smaller WC and VFAI, but comprised more females and higher SES, FFMI, and SMMI scores based on the univariate analysis (Table 1). The results of the Pearson’s correlation analysis for the body composition indices are shown in Table 2. The variance inflation factor (VIF) and collinearity diagnostics demonstrated a good model fit without any remarkable collinearity problem (VIF < 10, data not shown).

**Table 1:** Baseline demographic characteristics of the subjects between (N = 1,199).

	Survival	Non-survival
Total	1095 (91.33)	104 (8.67)**
Age	58.54 ± 10.78	72.16 ± 9.35**
Age > = 65	342(31.2)	86(82.7)**
Women	636(58.1)	40(38.5)**
High SES	365(33.3)	21(20.2)*
History of smoking	177(16.2)	30(28.8)*
History of alcohol consumption	154(14.1)	19(18.3)
History of habitual exercise	233(21.3)	21(20.2)
CCI	0.80 ± 1.30	1.70 ± 1.71**
BMI	24.68 ± 3.55	23.98 ± 3.59
WC	83.78 ± 9.83	86.71 ± 9.55**
PBF	31.52 ± 7.69	31.49 ± 8.83
FMI	7.92 ± 2.81	7.75 ± 3.04
FFMI	16.76 ± 2.11	16.22 ± 1.95*
VFAI	45.40 ± 12.57	53.41 ± 13.12**
SMMI	9.07 ± 1.31	8.78 ± 1.26*

Continuous data was analyzed using an independent t-test and was represented as mean ± SD. Dichotomous data was analyzed with a chi-square test and represented as N (percentile).

SES: socioeconomic status; CCI: Charlson comorbidity index; BMI: body mass index;

WC: waist circumference; PBF: percent body fat; FMI: fat mass index.

FFMI: fat free mass index; VFAI: visceral fat area index, and SMMI: skeletal muscle mass index.

*p < .05; **p < .001.

**Table 2:** Pearson’s correlation coefficients for the body composition indices.

	BMI	WC	SMMI	PBF	FMI	FFMI	VFAI
BMI	1						
WC	0.781**	1					
SMMI	0.544**	0.629**	1				
PBF	0.490**	0.204**	−0.384**	1			
FMI	0.745**	0.451**	−0.076*	0.936**	1		
FFMI	0.583**	0.635**	0.969**	−0.369**	−0.053	1	
VFAI	0.580**	0.526**	−0.001	0.628**	0.694**	0.038	1

BMI: body mass index; WC: waist circumference; SMMI: skeletal muscle mass index; PBF: percent body fat; FMI: fat mass index; FFMI: fat free mass index; VFAI: visceral fat area index; shaded numbers indicate a significant negative correlation.

*: *p* < .05; **: *p* < .001.

### The anthropometric parameters and all-cause mortality

The anthropometric parameters were used to evaluate the associations among BMI, WC, and PBF with all-cause mortality. The Cox proportional hazard model analysis adjusted according to age, gender, SES, CCI, and specific behaviors showed that a higher BMI and lower PBF were significantly associated with a lower risk of all-cause mortality (Table 3). In the model with the BMI and WC, the WC revealed a trend toward but not a significant association with all-cause mortality (Table 3).

**Table 3:** Cox proportional hazard model analysis of the association between the BMI, WC, and PBF and all-cause mortality (N = 1,199).

	HR (95% CI)	HR (95% CI)
Age	1.08 (1.06–1.11)**	1.07 (1.05–1.10)**
Gender (ref. Female)	1.48 (0.88–2.49)	2.58 (1.34–4.98)*
SES (ref. Low)	0.67 (0.39–1.14)	0.66 (0.38–1.14)
CCI	1.13 (1.01–1.26)*	1.13 (1.01–1.27)*
Smoking habit (ref. No)	0.99 (0.60–1.63)	1.01 (0.60–1.68)
Alcohol consumption (ref. No)	1.37 (0.78–2.43)	1.44 (0.82–2.55)
Exercise habit (ref. No)	0.87 (0.52–1.47)	0.87 (0.51–1.46)
BMI	0.92 (0.83–1.02)	0.90 (0.82–0.98)*
WC	1.02 (0.98–1.06)	–
PBF	–	1.06 (1.01–1.11)*

SES: socioeconomic status; CCI: Charlson comorbidity index; BMI: body mass index;

WC: waist circumference, and PBF: percent body fat.

*p < .05; **p < .001.

### The body composition indices and all-cause mortality

Using the body composition indices, we further analyzed the associations among the FMI, FFMI, SMMI, and VFAI with the risk of all-cause mortality using a Cox proportional hazard model analysis adjusted according to age, gender, SES, CCI, and specific behaviors. After adjusting using the generalized fat accumulation index (FMI), a higher FFMI was found to be significantly associated with a lower risk of all-cause mortality (Table 4). The adjustments with either FMI or the reginal fat accumulation index (VFAI) all revealed that a higher SMMI was significantly associated with a lower risk of all-cause mortality. Age, male gender, and CCI were found to be significantly positively associated with a higher risk of all-cause mortality in all of the analyses (Table 4).

**Table 4:** Cox proportional hazard model analysis of the association between the FMI, FFMI, SMMI, and VFAI and all-cause mortality (N = 1,199).

	HR (95% CI)	HR (95% CI)	HR (95% CI)
Age	1.07 (1.04–1.10)**	1.06 (1.03–1.09)**	1.10 (1.07–1.12)**
Gender (ref. Female)	2.81 (1.50–5.25)*	2.95 (1.49–5.87)*	2.04 (1.06–3.92)*
SES (ref. Low)	0.67 (0.39–1.16)	0.66 (0.38–1.14)	0.53 (0.32–0.89)*
CCI	1.13 (1.01–1.28)*	1.13 (1.01–1.27)*	1.17 (1.05–1.30)*
Smoking (ref. No)	1.00 (0.60–1.67)	1.08 (0.65–1.81)	1.51 (0.91–2.50)
Alcohol consumption (ref. No)	1.46 (0.83–2.57)	1.34 (0.75–2.38)	1.49 (0.85–2.62)
Habitual exercise (ref. No)	0.87 (0.52–1.47)	0.87 (0.52–1.46)	0.95 (0.57–1.57)
FMI	1.07 (0.98–1.16)	–	1.02 (0.94–1.11)
FFMI	0.81 (0.70–0.92)*	–	–
VFAI	–	1.01 (0.99–1.04)	–
SMMI	–	0.69 (0.53–0.90)*	0.71 (0.55–0.91)*

SES: socioeconomic status; CCI: Charlson comorbidity index; FMI: fat mass index.

FFMI: fat free mass index; VFAI: visceral fat area index, and SMMI: skeletal muscle mass index.

*p < .05; **p < .001.

### Analysis of older people

When considering interactions with older subjects more than 65 years of age, the FFMI and SMMI showed negative associations with a risk of all-cause mortality exclusively in elderly people. Impressively, the SMMI was associated with a higher risk reduction for all-cause mortality in comparison with the FFMI for the older subjects (Table 5).

**Table 5:** Cox proportional hazard model analysis of the associations between the FMI, FFMI, SMMI, VFAI, and all-cause mortality by age.

	Age < 65 (N = 771)			Age > = 65 (N = 428)		
Gender (ref. Female)	7.00 (1.48–33.01)*	4.87 (0.87–27.14)	6.13 (1.37–27.43)*	2.55 (1.28–5.09)*	3.23 (1.57–6.64)*	2.58 (1.28–5.17)*
SES (ref. Low)	0.71 (0.22–2.34)	0.72 (0.22–2.38)	0.46 (0.15–1.44)	0.93 (0.50–1.72)	0.86 (0.46–1.61)	0.71 (0.40–1.27)
CCI	1.44 (1.02–2.04)*	1.42 (1.01–1.99)*	1.33 (0.99–1.79)	1.14 (1.00–1.29)*	1.12 (0.99–1.27)	1.17 (1.04–1.31)*
Smoking (ref. No)	2.18 (0.55–8.73)	2.04 (0.52–8.00)	1.15 (0.33–3.94)	1.06 (0.59–1.89)	1.19 (0.67–2.11)	1.96 (1.12–3.41)
Alcohol consumption (ref. No)	1.00 (0.26–3.78)	0.98 (0.25–3.82)	0.86 (0.24–3.09)	1.40 (0.74–2.64)	1.21 (0.63–2.32)	1.42 (0.75–2.67)
Habitual exercise (ref. No)	0.56 (0.14–2.20)	0.66 (0.18–2.45)	0.72 (0.21–2.53)	0.96 (0.55–1.69)	0.93 (0.53–1.63)	1.05 (0.61–1.81)
FMI	1.16 (0.97–1.37)	–	1.08 (0.91–1.27)	1.08 (0.97–1.19)	–	1.03 (0.94–1.13)
FFMI	0.77 (0.56–1.07)	–	–	0.75 (0.64–0.87)**	–	–
SMMI	–	0.77 (0.43–1.37)	0.79 (0.49–1.28)	–	0.56 (0.42–0.75)**	0.52 (0.39–0.69)**
VFAI	–	1.02 (0.98–1.07)	–	–	1.02 (1.00–1.05)	–

Data was represented as: HR (95%CI).

SES: socioeconomic status; CCI: Charlson comorbidity index; FMI: fat mass index.

FFMI: fat free mass index; SMMI: skeletal muscle mass index, and VFAI: visceral fat area index.

*: *p* < .05; **: *p* < .001.

### Analysis of gender effects

The subgroup analysis by gender revealed that a higher FFMI and SMMI were significantly associated with a lower risk of all-cause mortality in the case of both genders, but a higher fat accumulation (FMI and VFAI) was found to be significantly associated with higher risk of all-cause mortality only in females (Table 6).

**Table 6:** Cox proportional hazard model analysis of the associations between the FMI, FFMI, SMMI, VFAI, and all-cause mortality by gender.

	Female (N = 676)			Male (N = 523)		
Age	1.08 (1.04–1.13)**	1.04 (0.99–1.10)	1.12 (1.08–1.17)**	1.06 (1.03–1.09)**	1.07 (1.03–1.11)*	1.07 (1.04–1.11)**
SES (ref. Low)	0.39 (0.09–1.71)	0.41 (0.09–1.80)	0.28 (0.07–1.16)	0.86 (0.45–1.61)	0.78 (0.41–1.47)	0.68 (0.38–1.22)
CCI	1.05 (0.85–1.31)	1.09 (0.87–1.36)	1.17 (0.94–1.45)	1.20 (1.04–1.39)*	1.18 (1.03–1.36)*	1.16 (1.02–1.31)*
Smoking (ref. No)	2.30 (0.29–18.07)	2.43 (0.31–19.02)	4.05 (0.54–30.49)	0.95 (0.55–1.64)	1.02 (0.59–1.75)	1.52 (0.91–2.55)
Alcohol consumption (ref. No)	–	–	–	1.63 (0.90–2.95)	1.47 (0.80–2.70)	1.49 (0.83–2.67)
Habitual exercise (ref. No)	0.79 (0.30–2.08)	0.88 (0.34–2.29)	0.64 (0.25–1.66)	0.78 (0.41–1.50)	0.80 (0.42–1.52)	1.06 (0.58–1.95)
FMI	1.23 (1.08–1.40)*	–	1.15 (1.02–1.30)*	0.95 (0.83–1.08)	–	0.91 (0.81–1.03)
FFMI	0.71 (0.54–0.93)*	–	–	0.82 (0.70–0.97)*	–	–
SMMI	–	0.62 (0.39–0.99)*	0.64 (0.38–1.06)	–	0.72 (0.52–0.99)*	0.68 (0.50–0.91)*
VFAI	–	1.05 (1.01–1.09)*	–	–	0.98 (0.95–1.02)	–

Data was represented as: HR (95%CI).

SES: socioeconomic status; CCI: Charlson comorbidity index; FMI: fat mass index.

FFMI: fat free mass index; SMMI: skeletal muscle mass index, and VFAI: visceral fat area index.

*p < .05; **p < .001.

## Discussion

This cohort study showed that the population with a higher BMI had a lower all-cause mortality rate, but there was a higher all-cause mortality rate in those with a higher PBF. The negative associations between FFMI and SMMI and all-cause mortality had been demonstrated in advance. These findings emphasize the critical predictive role of the body composition indices in all-cause mortality, especially in an older population.

The obesity paradox has been observed in many conditions, such as hemodialysis^18^, heart failure, and coronary heart disease^19^, as well as in older adults^20^. In our study, the fact that the BMI was significantly negatively associated with all-cause mortality highlights the fact that the obesity paradox may also exist in a community-based general population. The pathophysiology underlying this phenomenon has been discussed, including survival effects, but the findings have been inconclusive^20^. Consistent with our findings, some studies have suggested that the obesity paradox may be explained by specific body composition indices, such as fat mass and lean mass^21^. Previous studies have also concluded that the appendicular skeletal mass, SMMI, or FFMI may mediate associations between the BMI and adiposity and may be inversely associated with mortality, which makes them the best indices for prediction of mortality^22,23^.

The findings of the present study also demonstrated that FFMI and SMMI play a protective role in mortality in a population 65 years of age and older. A longitudinal cohort study on an older Japanese population revealed the FFMI and SMMI to be associated with mortality in a dose-dependent manner^24^. In a study on disabled nursing home residents, it was also concluded that low FFMI and SMMI are positively associated predictors of mortality^25^. Although, no significant associations among FM, SMM, and SMMI and mortality have been found in peritoneal dialysis patients^26^, a loss of skeletal muscle density was found to be significantly associated with increased mortality in pancreatitis patients^27^ and in older people with sepsis^28^. That is, with the exception of the BMI, the other body composition indices, especially the SMMI, may play a crucial role in all cause-mortality, especially in older individuals. Therefore, our findings provide information on the optimal body composition indices to use for health recommendations in clinical practice.

The mechanism by which the SMMI is protective is worth discussing. The severity of sarcopenia with decreased muscle mass, strength and/or function has been proven to be both a marker and predictor for health status and outcomes^29^. Chronic inflammation is involved in the pathogenesis of chronic diseases with insulin resistance, atherosclerosis, neurodegeneration, and tumor growth^30^. Contracting skeletal muscles can release myokines, a group of muscle-derived peptides with anti-inflammatory and endocrine effects, mediating direct anti-inflammatory effects and/or specific effects on visceral fat^31^. Decreased muscle mass can act as a marker indicating decreased physiologic reserves because it is associated with immune functions^32^, glucose disposal, protein synthesis, and mobility^33,34^. Therefore, skeletal muscle atrophy reflected in the form of a lower SMMI is associated with impaired cytokine^31^ and insulin signaling^35^ that may result in glucose intolerance, an expanded burden of chronic diseases, and hence, an increased risk of mortality. In the present study, exercise habits were also considered as a confounding factor in the model in an effort to provide additional information. In addition, a lower FFMI score is known to be associated with all-cause mortality in some diseases including cancer and chronic obstructive pulmonary disease^36,37^, as confirmed by the results of the present study. The potential mechanisms linking lower FFMI to higher mortality are systemic inflammation and low muscle mass related to lower physical activity^38^ as mentioned above.

It is noteworthy that some study results suggested a gender difference between body composition indices and mortality. Many previous studies have reported an inverse relationship between the FFMI, SMM and mortality risk in males^39,40^, but this association in women was found to be weaker or absent altogether^40,41^. In contrast, fat mass was found to be protective in older women but not in men^41^. However, the protective effects of body fat may be attributed to confounding factors related to pre-existing chronic diseases^41^. Other studies also report a stronger positive association between fat mass and cardiovascular diseases and all-cause mortality in both sexes with minor differences^17^. In this study, after adjusting for the CCI, the FMI and VFAI were found to be positively associated with all-cause mortality only in females. Increased VFA levels are a strong risk factor for metabolic syndrome in postmenopausal women^42^. In addition, carotid intima-media thickness has been shown to be correlated with VFA levels^43^. However, no significant associations between visceral adipose tissue and mortality in men or women were found in a colon cancer population^16^. These studies suggest that central obesity or fat distribution may play a pathophysiological role in all-cause mortality^15^. The possible mechanisms for differences between both genders in the association of FMI, VFAI and mortality may be due to sex-related different inflammation levels^44^ or the sex hormones^45^. The above-mentioned results highlight the fact that race, gender, and age differences may contribute to various relationships among body composition indices and the risk of all-cause mortality. Although interactions among age, gender, and mortality were demonstrated in this study, more studies are warranted to clarify these findings and to explore the mechanisms by which this association occurs in the future.

This study has both strengths and limitations. To the best of our knowledge, this is the first study using a community cohort with comprehensive follow-up data from the NHIRD to evaluate the role of body composition indices in all-cause mortality. It should be noted that we used the BIA method to measure body composition but not the DXA. However, both the multi-frequency BIA and DXA are comparable methods that can be used for measuring body composition, and both are recommended, especially in community-based populations^46^. The health-related behaviors (such as smoking and alcohol consumption) and the CCI may be changed during the follow-up period. We used stratified Cox model analysis by stratifying CCI to reconfirm those inferences. The trends of HRs between BMI, PBF, FMI, FFMI, VFAI, SMMI and all-cause mortality remain consistently (data not shown). However, the inferences of these risk factors on all-cause mortality are warranted for further study. Finally, all participants in the present study were Taiwanese, suggesting that generalizing our findings to other ethnicities living in different regions should be done with caution.

In conclusion, after considering all major confounders, the SMMI and FFMI were shown to be significantly negative predictors for all-cause mortality in an adult population, especially in an older population. Meanwhile, a higher fat mass or visceral fat distribution was shown to predict higher mortality in females.

## Materials and methods

### Participants

A total of 2,208 subjects were selected using a step by step, stratified systemic cluster sample of households, where two townships in Yun-Lin County (Douliou and Kukeng) were randomly selected in the first sampling step. Six Lis in Douliou (one Li from each district) and 3 villages in KuKeng were chosen. One in every five houses was selected, and all the residents who satisfied the inclusion criteria were invited to participate in this study. A final cohort population of 1,200 was enrolled from Yun-Lin County in central western Taiwan for 2009 and 2010^47,48^. Subjects who had been hospitalized in the prior 6 months, had congestive heart failure, renal failure, and those who were pregnant were excluded. This study was approved by the Institutional Review Board of National Cheng Kung University Hospital (A-ER-109–407), and informed consent was obtained from each subject, which was approved by the Institutional Review Board of National Cheng Kung University Hospital (IRB number: ER-98–084) in 2009. All methods were performed in accordance with the relevant guidelines and regulations.

### Measures and questionnaires

With the participants wearing light clothing and no shoes, body weight (BW, to the nearest 0.1 kg), and body height (BH, to the nearest mm) were all measured (Digital Eye-Level with 750; Detecto Scale, Webb City, MO, USA), and the body mass index (BMI, kg/m^2^) of the participants was then calculated. Standing naturally, looking forward, and wearing only their underwear, the participants’ WCs midway between the lateral lower rib margin and the superior anterior iliac crest to the nearest mm were measured by well-trained staff using a standard tape measure (Gulick II 67,019; Country Technology, Gays Mills, WI, USA) at the end of a gentle expiration phase^49^. After overnight fasting with an empty bladder, a validated multi-frequency bioelectrical impedance analysis (BIA) device (InBody 720; Biospace Co. Ltd., Seoul, Republic of Korea) was used to measure the body composition of each participant^42,46,50^. InBody (720) takes readings from the body using an eight-point tactile electrode method, measuring resistance at five specific frequencies and reactance at three specific frequencies. Total body water (TBW) was estimated from area, volume, length, impedance, and a constant proportion (specific resistivity). The FFM was estimated by dividing TBW by 0.73. Percent body fat was calculated using the FM divided by the BW^51^. Skeletal muscle mass (SMM) was estimated using the Janssen's equation: SMM = ([Ht^2^/R × 0.401] + [sex × 3.825) + (age ×  − 0.071]) + 5.102, where height was measured in cm, and the resistance was measured in ohms^52^. Visceral fat area (VFA), defined as a cross sectional area of visceral fat in the abdomen at the umbilical level, as measured using a BIA, correlated significantly with that acquired using CT (r = 0.922 for VFA)^53^. The FM, FFM, SMM, and VFA were then divided by body height squared to obtain the fat mass index (FMI), fat free mass index (FFMI), skeletal muscle mass index (SMMI) (kg/m^2^), and visceral fat area index (VFAI), respectively. A baseline questionnaire was used to collect information about lifestyle and habits, including exercise, smoking, and alcohol consumption, socioeconomic status, past medical conditions, and number and types of medications. A current smoker was defined as a subject who had smoked 1 pack (20 cigarettes) at least per month for more than 6 months and was still smoking. An alcohol drinker was defined as a subject who had consumed alcohol more than once a week for more than six months before the study. Habitual exercise was defined as those who engage in exercise more than three times per week^54^. The socioeconomic status score was calculated according to a modified Hollingshead’s index of social position and was categorized as high (levels 4 and 5) and low (levels 1–3)^47^.

### NHIRD data

The National Health Insurance (NHI) of Taiwan, a globally unique program in force since 1995, manages more than 99.9% of the medical claims data of all citizens, keeping records in the National Health Insurance Research Databank (NHIRD)^55^. This databank has been used as an important source of data for evidence-based studies in Taiwan. All our participants were included in Taiwan ‘s NHIRD at some point during our observation period (2008 to 2017), and the CCI was then calculated based on the participant’s disease records^56^. We chose the CCI as the comorbidity measurement because it is a strong predictor of outcome in chronic disease patients, and the CCI scores using the international classification of disease (ICD) code information have been proven to have a significant association with mortality^57,58^. The primary outcome was the first claim of death up to December 31, 2018 in Taiwan’s NHIRD. All claims records of deaths occurring from 2008 to 2018 in the NHIRD were identified. The cumulative all-cause mortality rates were obtained for all participants at the end of the follow-up period. Finally, a total of 1,199 participants were followed completely from their examination date (2009–2010) until either death occurred or until December 31, 2018.

### Statistical methods

The data analysis for this paper was generated using SAS software, Version [9.4] of the SAS System for Windows. The basic demographic data for our study participants were analyzed using the mortality data reported by the Taiwan NHIRD. Intergroup comparisons of basic demographic data between the survival and non-survival cohorts were performed using a chi-square test for the categorical variables and the Student’s t-test for the continuous variables. Interrelationships were analyzed using a Pearson’s correlation analysis to evaluate the correlations between the body composition indices. Collinerity was analyzed using the variance inflation factor and collinearity diagnostics to access the model fitness. To determine the individual contributions of the different body composition indices, a Cox proportional hazard model analysis was applied to assess the association between these indices and the risk of all-cause mortality, as adjusted for age, gender, exercise, smoking, alcohol consumption, SES, and CCI. The variates in the models selected for the analysis were based on whether they were previously mentioned as key factors, any conflict of correlation or collinearity of the variables in the model, or could be classified as factors of interest. Several models were analyzed, and the results didn’t change to any significant degree. All the anthropometric indices were divided by height squared for the purpose of assessing muscle or fat mass, as recommended by international study groups^59^. Because age and gender are important determinants of mortality, an analysis was performed separately on two age and gender subgroups according to the dichotomized cutoff point between young (those aged between 40–65 years) and older (those aged 65 years or more) individuals. Significance was set at *p* < 0.05 (two-tailed).

## Abbreviations

BMI: Body mass index
WC: Waist circumference
FM: Fat mass
PBF: Percent body fat
FMI: Fat mass index
FFMI: Fat free mass index
SMMI: Skeletal muscle mass index
VFAI: Visceral fat area index
NHI: National Health Insurance
NHIRD: National Health Insurance Research Databank
